# Heavy Metal Levels in Milk and Cheese Produced in the Kvemo Kartli Region, Georgia

**DOI:** 10.3390/foods10092234

**Published:** 2021-09-21

**Authors:** Rami Al Sidawi, Giorgi Ghambashidze, Teo Urushadze, Angelika Ploeger

**Affiliations:** 1 Specialized Partnership in Sustainable Food Systems and Food Sovereignty, Faculty of Organic Agricultural Sciences, University of Kassel, 37213 Witzenhausen, Germany; a.ploeger@uni-kassel.de; 2 School of Agricultural and Natural Sciences, Agricultural University of Georgia, Tbilisi 0159, Georgia; g.ghambashidze@agruni.edu.ge (G.G.); t.urushadze@agruni.edu.ge (T.U.); 3 Scientific-Research Centre of Agriculture, Ministry of Environmental Protection and Agriculture of Georgia, Tbilisi 0159, Georgia

**Keywords:** heavy metal, milk, cheese, Georgia

## Abstract

Milk and dairy products are among the most important food sectors in Georgia, and milk is considered one of the most essential foods in the human diet according to Georgian food culture. Kvemo Kartli is one of the major regions in Georgia for milk production. This region suffers from heavy metal contamination in soil and water because of the mining industry. This study was conducted to determine the concentrations of cadmium, lead, iron, zinc, copper, chromium, manganese, cobalt, nickel, selenium and molybdenum in milk and cheese and to evaluate whether the concentrations of these elements correspond to the permissible levels of toxic elements in milk and cheese for Georgia and the EU. In total, 195 milk samples and 25 cheese samples (16 from Imeruli cheese and nine from Sulguni cheese) were collected from nine different villages in the Kvemo Kartli region in Georgia: Chapala, Vanati, Bolnisi, Mtskneti, Sabereti, Ratevani, Khidiskuri, Kazreti, Kvemo Bolnisi. The determination of heavy metal in all samples was carried out by inductively coupled plasma-mass spectrometry. The research results show that the concentration of these elements in most milk samples is fairly constant for all villages and is less than the permissible levels, except for seven samples from the following villages: Kvemo Bolnisi, Bolnisi, Mitskineti and Ratawani, where the concentration of lead in the milk samples was higher than the permissible limits mentioned in the literature, ranging from 0.027 to 1003 mg L^−1^. As for copper, its concentration in milk in Sabereti and Vanati villages was above the permissible limits according to the EU limit, ranging from 0.42 to 1.28 mg L^−1^. For cheese samples, the concentration of cadmium, lead, copper, Co and Ni in the two types of cheese was less than the permissible limit according to the laws of Georgia. Finally, the heavy metal concentrations in Imeruli and Sulguni cheese for manganese (Mn), chromium (Cr), selenium (Se), molybdenum (Mo) zinc (Zn) and iron (Fe) were above the permissible limit. Thus, the study results showed that the consumption of milk does not pose a direct and serious threat to the health of consumers. As for the two types of cheese, future studies and continuous monitoring are necessary to assess the cheese content of trace elements and the risk of its consumption to the consumer.

## 1. Introduction

Milk and dairy products contain vital nutrients [1,2]. In addition to proteins, dairy products contain vitamins, lactose, unsaturated fatty acids and many minerals. On the other hand, they may contain amounts of various toxic pollutants. Many plants such as Oonopsis, Xylorrhiza and others accumulate selenium, and through grazing, these plants may lead to livestock intoxication [3]. Other toxic components come from contaminated soil (from industrial activity) or are geogenic, which pose a significant risk to human health, too [4,5]. 

The mineral contents of milk and dairy products can be classified into basic elements (iron, copper and zinc), present in low doses. These trace metals in milk are more dangerous than in other foods since milk consumption is higher in the most vulnerable age groups (the elderly and infants), approximately 30–150 kg/person/year in general [6]. As for unnecessary or toxic elements (such as lead, cadmium and others), the latter’s presence, even in low concentrations, may lead to serious health problems in humans [1,2,7,8,9]. Some of the side effects of heavy metal on the human body are kidney failure, genetic mutations and nervous system disorders. It can also cause cardiovascular problems, many types of cancers, respiratory disorders, a weak immune system and it can also cause infertility [7,10,11]. 

The human body may be exposed to heavy metal in several ways: consuming contaminated drinking water, inhaling dust or transferring these minerals from polluted soil or groundwater to plants. Furthermore, the mobility of heavy metals depends on a considerable number of factors, and the level of absorption and accumulation of these trace elements in plants may vary according to soil type, micronutrient content, moisture and pH [12,13]. By directly consuming contaminated food plants, heavy metals may easily be transmitted to animals, as livestock feed on the grass in pastures or on concentrated feeds, which are contaminated, transfer these minerals to the animals. However, the transfer of these minerals can vary highly, especially in cattle [14,15,16,17,18,19,20,21].

For example, one study on traditional farms in central Greece analyzed the level of heavy metal in the bodies of cows and sheep who were exposed to fodder contaminated with heavy metal by industrial areas and crowded roads close to agricultural lands. The results showed that through foraging, copper transfers to the cows’ bodies to the liver was in a much higher concentration than the level of this element in each of the cows’ kidneys and muscle tissues; whereas the ratio of copper carryover to milk was very low or absent. The results also showed the presence of Cu in cow manure was the same as that found in cow kidneys and muscle tissues [22].

In another study conducted in Ibadan, Nigeria, on sites contaminated with lead slag, four heavy metal, Pb, Cd, Cu and Zn, were analyzed in the grass samples of the area where livestock grazed. The results showed that plants and feed contained all four heavy metal, and a lead concentration above the permissible limits was discovered. The concentrations of zinc, cadmium and copper were within the recommended limits. In that study, milk, faeces and blood of cows grazing in the area were analyzed, and the presence of lead in milk, blood and faeces was higher than the detectable limit of the measuring devices [23].

Another study examining the extent of heavy metal transfer to cow’s milk proved that milk protein is affected by heavy metal contamination more than other milk components [24]. Cadmium in milk was associated with casein fractions, as 14% of cadmium was transferred to whey and 60% to curd [25]. Another study on copper, cadmium and lead in cheese and milk proved that the concentration of elements in cheese is much higher than in milk, as most of these elements are bound to casein, whereas just a small amount was released in whey [25]. A study on cheese showed that adding coagulant and salt may increase lead, cadmium and copper concentration in the curd [25].

The production of milk and dairy products in Georgia is considered one of the most important sectors in the country’s food industry, and Kvemo Kartli is one of the most important areas that produce dairy and cheese in Georgia [26,27,28]. A popular type of cheese, Sulguni and Imeruli (Appendix B), is mainly made in this region, where its sale and consumption are still local and national, and it cannot be exported because it does not meet international standards [29,30,31,32]. However, this region suffers from many problems, namely environmental pollution [33]. Part of the cause for pollution began in 1975, near the Kazreti village, when the “Madneuli” mining plant started its operations [34]. In 2014, in the Dmanisi-Bolnisi area of Kvemo Kartli, on the right and left banks of the Mashavera River, the RMG gold and copper mine was opened with government approval [34,35,36,37,38,39]. As a result of factory activities, severe environmental pollution with toxic metals has appeared. From this point of view, previous studies and research in this region have shown trace metal contamination of rivers, particularly the Mashavera River Basin, which was counted as one of several rivers contaminated with heavy metal in Georgia [33,34,40,41,42,43].

Farmers in this region depend heavily on the Mashavera River’s water, which they use to irrigate their crops and lands, and some farmers may also use it as drinking water [44]. The Kvemo Kartli region is of great importance for its prominent participation in agriculture production. The river region supports all sectors producing for the local diet, such as beans, corn and wheat crops, dairy and meat products, and many types of vegetables and many kinds of local wine. Farmers also use the water and grass for grazing cattle, goats and sheep [44,45]. Numerous studies and research in recent years have proven that the amount of water and soil pollution from heavy metal is vast in this area and has exceeded the permissible and highly accepted standards [34,36,46,47]. Therefore, the pollution of rivers, soil and air with heavy metal will affect the health of the population on the one hand and the local diet in Georgia on the other hand [34,45].

After studies were previously conducted to determine the extent of contamination of rivers, soil and plants with heavy metal, the main objective of this research paper is to determine the level of heavy metal in raw milk and cheese manufactured in the Kvemo Kartli area.

## 2. Methodology

### 2.1. Sampling Sites

**Figure 1:**
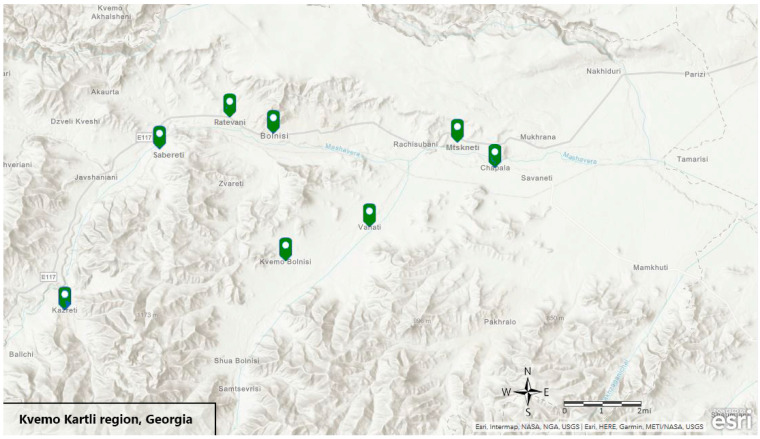
Map of the study area (Authors’ illustration).

A total of **195** samples of cow’s milk and 25 samples of Imeruli and Sulguni cheese were collected during summer 2019 in nine different villages in the Kvemo Kartli region in Georgia: Chapala, Vanati, Bolnisi, Mtskneti, Sabereti, Ratevani, Khidiskuri, Kazreti and Kvemo Bolnisi (Figure 1).

The sampling sites are located in villages adjacent to the Mashavera river or one of its sub-channels proven to be contaminated with heavy metal. The sampling protocols of milk, Imeruli cheese and Sulguni cheese were analyzed to reveal these elements: Cd, Pb, Cr, Cu, Fe, Mn, Se, Zn, Co, Ni, Mo.

### 2.2. Collection of Samples

All milk samples were collected in the evening when the cows returned from grazing, as most farmers milked their cows in the evening. Polyethylene containers washed with nitric acid (concentration: 65%) were used to collect all milk samples. They were placed in a cooler with ice packs and then transferred directly to the accredited state laboratory, where they were stored at a temperature of −20 °C until analysis. Several procedures were followed to mitigate potential external contamination during milk sample collection:

- no mechanical milking machines were used,
- the person collecting the samples wore nitrile or latex gloves,
- the udder was well ‘sanitized’ before milking,
- all samples were numbered according to the villages in which they were taken from farmers.

The cheese samples were subsequently taken from the same farms that allowed us to take the milk samples. All were processed-made homemade cheese, which is typically sold in local markets.

### 2.3. Preparation and Analysis of Samples

#### 2.3.1. Certified Reference Material (CRM)

Accuracy of obtained results was ensured by regular participation in Inter-Laboratory Proficiency Testing programs. Precision testing was carried out by performing duplicate analysis on one sample for every 20 samples. Calibration standards were prepared from CRM produced according to ISO 17034 and are traceable to NIST (National Institute of Standards and Technology).

The 10-point calibration curves (nine standards and one blank) were constructed for each element, and the correlation coefficients obtained were ≥0.9999 before starting the analysis. 

Daily analyses of internal control materials were used for controlling the repeatability and accuracy. Uncertainties of the performed measurements were calculated following the EURACHEM guideline [48].

#### 2.3.2. Milk and Cheese Samples

At first, 50.0 mL per sample in two replicates were placed in an open porcelain crucible treated with 1 mL of concentrated nitric acid (65%) and heated slowly on a hot plate (IKA, Tbilisi, Georgia) until fuming stop. As for cheese samples, 10.0 g per sample in two replicates were placed in an open porcelain crucible treated with 1 mL of concentrated nitric acid (65%) diluted (1:1) with deionized water, kept 15 min. Then both samples were ashed in a muffle furnace (Nabertherm, L15/11, Germany) preheated at 250 °C with a gradual increase of temperature (50 °C every 30 min) to reach 450 °C. Ashing continued until obtaining grey ash, the residues were dissolved with dilute nitric acid, and the mixture was slowly dried on a hot plate (IKA, Staufen, Germany) at 140 °C. After cooling, samples were placed back to the muffle furnace at 300 °C for 30 min. The last step was repeated until white ash was obtained. Finally, the ashed samples were dissolved with 5 mL of concentrated nitric acid and diluted to 25 mL (for cheese samples, diluted to 50 mL). With deionized water and filtered, two blank samples were carried out, in the same way using the reagents alone. The determination of metal contents in the obtained filtrate was carried out by inductively coupled plasma-mass spectrometry (ICP-MS, Agilent 7800, Agilent Technologies: Santa Clara, CA, USA) [49]. The LOD (limit of detection) for the studied metals are the following: for milk: Cr (0,00013), Mn (0,00010), Fe (0,00041), Co (0,00001), Ni (0,00007), Cu (0,00010), Zn (0,00033), Mo (0,00005), Cd (0,00001), Pb (0,00006) mg L^−1^. As for cheese, there are: Cr (0,0013), Mn (0,00033), Fe (0,00349), Co (0,00003), Ni (0,00034), Cu (0,00106), Zn (0,00092), Mo (0,00023), Cd (0,00024), Pb (0,00018) mg/kg [wet weight (ww)].

All chemicals used were analytical grade with high purity. All plastic and glassware were cleaned using diluted (1:1) nitric acid in a hot acid bath and rinsed with deionized water before use.

This analytical methodology was used according to: GOST 26929-94. Raw material and foodstuffs. Preparation of samples. Decomposition of organic matters for analysis of toxic elements, 1994; GOST 26929-94 is a Georgia national standard method for analysis of food, including milk and milk products. The laboratory is a state research institute, it participates in proficiency testing programs every year to perform external quality control. As a state institute, it is not accredited.

### 2.4. Data Analysis

The heavy metal values shown in this study are expressed as mean ± SD of the repeated measurement. SPSS software version 27.0 (IBM, Armonk, NY, USA), was used to obtain the descriptive statistical analysis, to calculate the mean and standard deviation; in addition, an independent samples *t*-test was used to determine whether the means of two independent samples were different.

## 3. Results and Discussion

### 3.1. Toxic Metals

Table 1 shows the average concentrations of heavy metal in fresh cow’s milk from nine villages in the Kvemo Kartli region in Georgia.

The contents of lead and cadmium in the milk samples are presented in Table 1. The concentrations of lead detected ranged from 0,004 to 0,048 mg L^−1^. As for cadmium (Cd) LOD: 0,00001 mg L^−1^, the results showed that its concentration in fresh raw milk was <0,001 mg L^−1^.

Comparing the lead and cadmium contents in milk samples with the maximum permissible limits (MPL) established by the International Dairy Federation (IDF) [50] and with the Maximum Permissible Concentrations (MPC) according to the Georgian regulation [51], the mean concentration of both cadmium and lead in raw cow’s milk samples were lower than MPL and MPC, respectively (Cd: 0.0026 mg L^−1^, Pb: 0.02 mg L^−1^ and Cd: 0,03 mg L^−1^, Pb: 0, mg L^−1^). Furthermore, according to the European Commission and Codex Alimentarius Commission, the limit for Pb in milk is 0.02 mg L^−1^ [52,53]. Thus, the analyzed milk samples did not exceed this permissible limit.

**Figure 2:**
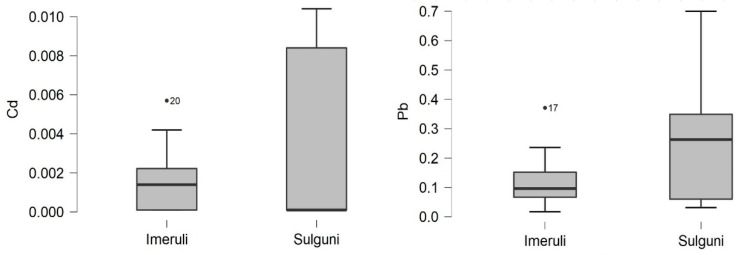
The cadmium (Cd) and lead (Pb) content of Imeruli and Sulguni cheese samples (mg/kg ww).

As for the cheese samples, Table 2 shows that the mean concentration of cadmium content of both types of cheese (Imeruli and Sulguni) was higher than in the milk samples (as expected), 0.002 and 0.007 mg/kg, respectively (Figure 2). However, it remains under the permissible limit. In the case of Pb, Sulguni cheese contained the highest concentration, 0.25 mg/kg ww, compared to Imeruli cheese, 0.12 mg/kg ww.

This difference between the cheese types may be due to the different methods used for cheese production; Imeruli cheese is usually taken as the base for making Sulguni cheese. To produce a kilogram of Imeruli cheese, one needs to have about seven liters of raw milk and only three ingredients: milk, salt and rennet. First, a solution of saltwater is prepared, and unheated curd is soaked in it for a period ranging from two to three days. The purpose of the brine is to stop the effect of bacteria, which enables the cheesemaker to adjust the time to control the acidity level of the Imeruli cheese, thus obtaining a highly melted cheese. Well-prepared Imeruli cheese should also have many small holes, as their presence is an indication that the bacteria have used up the lactic acid in the cheese, releasing carbon dioxide [54,55,56,57].

As for the manufacture of Sulguni cheese, either Imeruli cheese is used directly as the base or the following ingredients: milk, salt, rennet, whey and cream. About 10 L of raw milk are needed to produce a kilogram of Sulguni cheese. To prepare the Sulguni cheese, all these ingredients are combined and gently heated; the cheesemaker shapes it into a ball, then dried and pressed. Sulguni cheese, compared to other types of cheese, contains magnesium, phosphorus, potassium, sodium and iron [54,55,56,57].

According to the European Commission and Codex standards [53,58], it was observed that the Pb content in both cheese samples was above the maximum level (0.020 mg/kg ww). However, according to the Georgian regulation [51], the Maximum Permissible Concentrations (MPC) of lead in both types of cheese is less than the permissible limit (0.50 mg/kg ww).

This study showed that the cadmium concentration is very low and below the permissible levels, although several studies have confirmed that the water and pastures used by farmers in these regions are contaminated with heavy metal, including cadmium [39,47]. Cadmium and lead are considered highly toxic and have harmful effects on human health [59,60]. Milk usually contains a very low concentration of cadmium, so when there is a high cadmium content in the milk, the reason is that these animals may have fed on cadmium-contaminated feed or drank from contaminated water as well [61]. The acute toxicity of cadmium in the human body leads to the defection of the skeletal and cardiovascular systems [61,62].

**Table 1:** Essential trace elements and heavy metal in milk *n* = 195 (mean ± standard deviation; mg L^−1^) obtained in an area with water and pastures contaminated with heavy metal through mining industry in the Kvemo Kartli region, Georgia.

Mean Concentration (mg L^−1^) ± Standard Deviation (SD)											
Milk Samples	Trace Elements									Toxic Metals	
	Chromium (Cr)	Manganese (Mn)	Iron (Fe)	Cobalt (Co)	Nickel (Ni)	Copper (Cu)	Zinc (Zn)	Selenium (Se)	Molybdenum Mo	Cadmium Cd	Lead Pb
Bolnisi (*n* = 22)	0,002 ± 0,0016	0,036 ± 0,026	0,987 ± 0,841	<0,001	0,001 ± 0,0007	0,274 ± 0,370	2,975 ± 1,423	0,007 ± 0,0106	0,014 ± 0,0022	<0,001	0,006 ± 0,0079
Chapala (*n* = 22)	0,004 ± 0,002	0,075 ± 0,010	1,541 ± 1,284	0,0056 ± 0,003	0,017 ± 0,004	0,173 ± 0,079	3,458 ± 2,054	0,042 ± 0,023	0,022 ± 0,009	<0,001	0,008 ± 0,006
Daba Kazreti (*n* = 24)	0,002 ± 0,0036	0,044 ± 0,0617	1,391 ± 2,0387	0,004 ± 0,0107	0,002 ± 0,0016	0,133 ± 0,1404	2,411 ± 1,7129	0,005 ± 0,0038	0,009 ± 0,0068	<0,001	0,005 ± 0,0039
Kvemo Bolnisi (*n* = 24)	0,003 ± 0,002	0,036 ± 0,011	0,717 ± 0,525	0,003 ± 0,003	0,007 ± 0,005	0,133 ± 0,055	3,116 ± 0,959	0,020 ± 0,020	0,034 ± 0,025	<0,001	0,048 ± 0,204
Khidiskuri (*n* = 22)	0,001 ± 0,0013	0,023 ± 0,0229	0,502 ± 0,3815	0,007 ± 0,0172	0,006 ± 0,0075	0,120 ± 0,0811	2,223 ± 1,9752	0,020 ± 0,0142	0,011 ± 0,0092	<0,001	0,009 ± 0,0067
Mitskineti (*n* = 22)	0,003 ± 0,0014	0,049 ± 0,0185	1,089 ± 1,5586	0,005 ± 0,0030	0,002 ± 0,0026	0,142 ± 0,0544	3,916 ± 0,5227	0,006 ± 0,0022	0,022 ± 0,0138	<0,001	0,008 ± 0,0114
Ratawani (*n* = 23)	0,003 ± 0,002	0,058 ± 0,023	2,650 ± 2,137	0,002 ± 0,001	0,003 ± 0,003	0,404 ± 0,189	4,209 ± 1,671	0,011 ± 0,006	0,035 ± 0,025	<0,001	0,013 ± 0,015
Sabereti (*n* = 12)	0,002 ± 0,0006	0,032 ± 0,0059	5,537 ± 0,5251	<0,001	0,002 ± 0,0009	0,568 ± 0,1445	2,862 ± 0,3414	0,011 ± 0,0063	0,004 ± 0,0017	<0,001	0,004 ± 0,0029
Vanati (*n* = 24)	0,004 ± 0,0025	0,079 ± 0,0361	6,150 ± 2,5317	0,002 ± 0,0017	0,004 ± 0,0029	0,592 ± 0,2698	4,294 ± 1,0783	0,007 ± 0,0034	0,047 ± 0,0108	<0,001	0,012 ± 0,0115
Permissible limit *	0,02 [63] ^1^	0,02–0,05 [64] ^2^	0,7 [65,66] ^3,4^	0,006 [63,66,67] ^1,4,5^	0,027 [63,67] ^1,5^	0,4 [68] ^6,7^	3–5 [69] ^8^/2–6 [70] ^9^	0,5 [71] ^10^	0,05 [63] ^1^	0,2 [51] ^6^	0,020 [53,58] ^11,12^0,500 [51] ^6^
LOD, mg L^−1^	0,00013	0,00010	0,00041	0,00001	0,00007	0,00010	0,00033	0,00533	0,00005	0,00001	0,00006

*^1^. Flynn, A. (1992). ^2^. Knowles et al. (2006). ^3^. Storelli et al. (2007) ^4^. Safonov, V. (2020). ^5^. L. Hurleyw (1997). ^6^. Ministry of Labour and Health, and Social Affairs of Georgia. (2001) ^7^. European Commission (EC). (2001). ^8^. World Health Organization (1996). ^9^. Pechovà A, et al. (2008). ^10^. EFSA—European Food Safety Authority (2011). ^11^. European Commission. (2006). ^12^. Codex Alimentarius. (1995).

**Table 2:** Trace elements and toxic metals in Imeruli and Sulguni cheese *n* = 25 (mean ± standard deviation; mg/kg ww) were obtained in an area with water and pastures contaminated with heavy metal (caused by mining industry) in the Kvemo Kartli region, Georgia.

Mean Concentration (mg/kg *) ± Standard Deviation (SD)			
	Cheese Samples *n* = 25		
Trace Elements	Imeruli (*n* = 16)	Sulguni (*n* = 9)	LOD
Cr	0,035 ± 0,017	0,079 ± 0,057	0,0013
Mn	0,886 ± 0,595	2,348 ± 2,267	0,00033
Fe	69,09 ± 64,918	101,1 ± 91,166	0,00349
Co	0,013 ± 0,011	0,03 ± 0,026	0,00003
Ni	0,011 ± 0,007	0,026 ± 0,029	0,00034
Cu	1,261 ± 0,739	2,463 ± 2,314	0,00106
Zn	75,86 ± 52,528	124,8 ± 97,775	0,00092
Se	1,003 ± 0,901	3,06 ± 3,144	0,01107
Mo	0,289 ± 0,111	0,401 ± 0,254	0,00023
**Toxic metals**			
Cd	0,002 ± 0,0015	0,007 ± 0,003	0,00024
Pb	0,121 ± 0,093	0,258 ± 0,215	0,00018

* Results are presented on a wet weight (ww) basis.

As for Pb, the results show that the content in milk was low compared to cheese, as it was higher in the two types of cheese than the maximum level according to European standards. The results may be cause for concern since Pb is very dangerous to human health. Exceeding the permissible levels may have carcinogenic effects, and it may cause direct genotoxicity or an increase in oxidative stress, expression of growth factors and altered DNA repair [72]. However, the Pb values in this study are considered below the established Georgian standards.

The Sulguni cheese contained the highest amount of Pb compared to the Imeruli cheese. One reason may be the difference in the methods and making of these two types of cheese. It is known that during cheesemaking, the hydrolysis of ĸ-casein causes milk to be divided into two compounds: (1) the curd, which is mainly composed of casein and fat; (2) the whey, containing all soluble compounds, the most abundant of which are lactose and whey proteins. It is worth highlighting that, among heavy metals, lead tends to associate to casein more than to whey proteins, which contributes to an increase of its concentration in cheese. On the other hand, the moisture content in cheese is very important, as, with higher water content, the proportion of Pb in cheese (wet weight) is diluted [73].

### 3.2. Trace Elements

**Figure 3:**
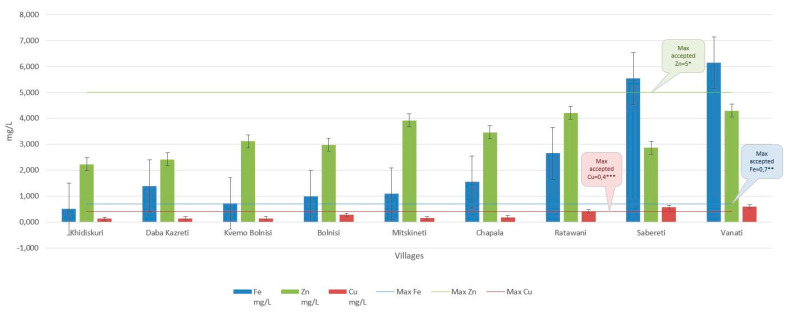
Concentration ranges (mg L^−1^) of Iron, zinc and copper in milk samples (N _Bolnisi_ = 22, N _Chapala_ = 22, N _Daba Kazreti_ = 24, N _Kvemo Bolnisi_ = 24, N _Khidiskuri_ = 22, N _Mitskineti_ = 22, N _Ratawani_ = 23, N _Sabereti_ = 12, N _Vanati_ = 24). * Maximum permissible limits for Zn, ** Maximum permissible limits for Fe, *** Maximum permissible limits for Cu.

**Figure 4:**
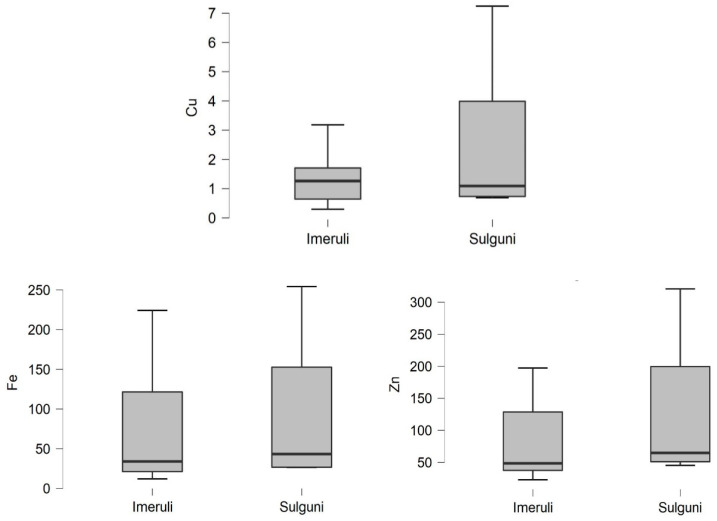
The iron, zinc and copper content of Imeruli (*n* = 16) and Sulguni (*n* = 9) cheese samples (mg/kg wet weight).

Furthermore, Table 1 and Figure 2 show the zinc, copper and iron concentrations in the raw milk and cheese samples. The concentrations of these trace elements (Zn, Fe and Cu) were detected in milk samples in amounts ranging from 2.22 to 4.29 mg L^−1^ (Zn), 0.5 to 6.15 mg L^−1^ (Fe) and 0.12 to 0.59 mg L^−1^ (Cu), respectively (Figure 3), and in both cheese samples Imeruli, 75.86 mg/kg ww Zn), 69.09 mg/kg ww (Fe) and 1.261 mg/kg ww (Cu), and Sulguni 124.8 mg/kg ww (Zn), 101.1 mg/kg ww (Fe) and 2.463 mg/kg ww (Cu), respectively (Figure 4).

According to WHO, the permissible limit for zinc in raw milk should be between 3–5 mg L^−1^ [69]. Compared to Pechová et al. the permissible zinc content in milk could be within 2–6 mg L^−1^ [70]. Thus, the zinc content in raw milk in this study is considered within the acceptable limit. Comparing the amount of zinc in the milk samples with the cheese samples shows that the Imeruli and Sulguni cheese (mg/kg WW) zinc content is much higher than the concentration in the milk (75.86, 124.8), respectively, where the processing of cheese affects that.

Zinc is considered a heavy metal due to its high density (7.133 g/cm^3^); once it exceeds 5 g/cm^3^, it is considered a heavy metal [74]. Still, zinc has an important role in the immune system’s physiological processes and functional performance in the human body. It is also involved in the structure and activity of approximately 300 enzymes in the body. These enzymes are responsible for replication and insulin secretion and cellular differentiation, protein synthesis and nucleic acid, and sexual maturation [75,76]. On the other hand, an increase in zinc content in the body and chronic exposure to it has negative effects on the human body, leading to anaemia and leucopenia and causes gastrointestinal diseases and diarrhea [75,76].

As for copper, the European Union has set the highest permissible amount of copper in milk and its products, which should not exceed 0.4 mg L^−1^ [68]. The results show that the concentration of copper in milk was less than the permissible limit, except for the Sabereti and Vanati regions, where the concentration of copper in milk was higher than the permissible value (0.56 and 0.59 mg L^−1^), respectively.

As for the cheese samples, the copper content in both types of cheese, Imeruli and Sulguni, and according to the Georgian regulation MPC [51] was less than the permissible limit (1.26 and 2.46 mg/kg wet weight), respectively. Copper is also considered one of the essential elements for humans but exceeding the normal permissible consumption levels may lead to toxic effects on the human body. It may cause gastrointestinal disorders, liver cirrhosis, reduced immunity, neurological disorders and dermatitis [65,77,78]. 

As for iron, the results in Table 1 and Table 2 show that iron concentrations in both milk and cheese were higher than the permissible limit [66,79] (0.138–0.700 mg/kg WW) in all the villages except for Khidiskuri village. The amount of iron in milk in this village was less than the permissible limit (0.502 mg L^−1^). In addition, the analysis of milk samples shows that the concentration of iron was relatively high in raw milk in the villages of Sabereti and Vanati compared with the rest of the villages (5.53 and 6.15 mg L^−1^), respectively.

The iron content of both Imeruli and Sulguni cheese samples was very high compared to the raw milk samples (69.09 and 101.1 mg/kg wet weight), respectively. Table 2 shows that Sulguni cheese had a higher iron content than Imeruli cheese, as the concentration of each of them exceeded the permissible limit.

Iron is an essential and important element for the human body, as it participates in many redox reactions [80]. Iron also has critical metabolic functions, and one of its most essential functions is oxygen transport. Iron deficiency in the body leads to decreased immune function, anaemia, impairment of cognitive performance and psychomotor development [80]. An increase in iron in the body may lead to dysfunction of the liver, spleen and brain [81]. In addition, iron can influence the concentration of other minerals in the body, as it can help increase cobalt and decrease calcium, copper and chromium (through excretion or binding) [81].

**Figure 5:**
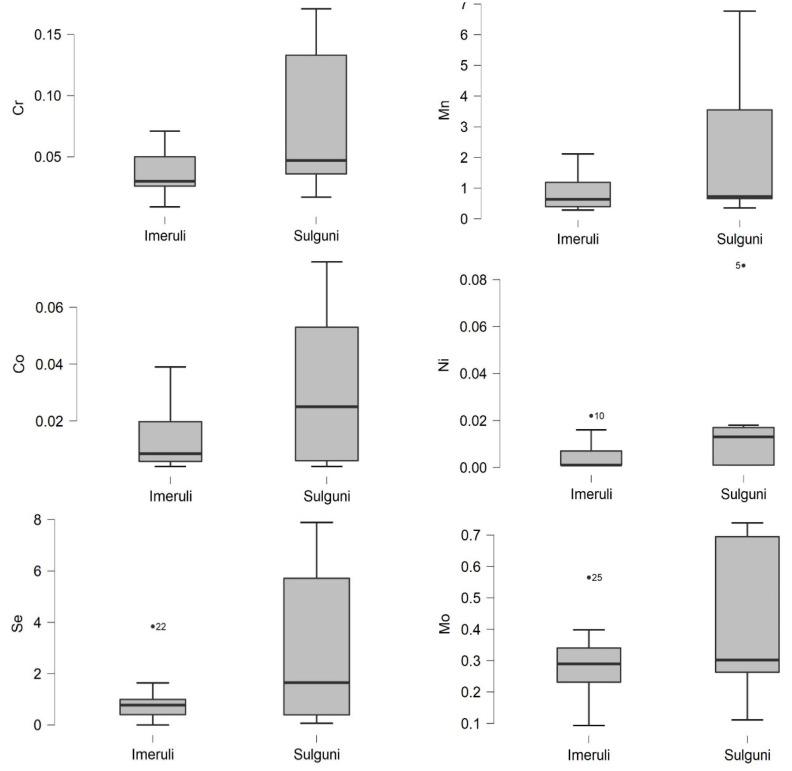
The Cr, Mn, Co, Ni, Se and Mo content of Imeruli and Sulguni cheese samples (mg/kg wet weight).

Furthermore, and based on the results in Table 1 and Table 2, the highest concentrations of the essential trace elements in the raw cow’s milk samples in the order of Cr, Mn, Co, Ni, Se and Mo (mg L^−1^) were detected in raw milk in amounts ranging from 0.001 to 0.004, 0.023 to 0.079, 0.001 to 0.007, 0.001 to 0.017, 0.004 to 0.042 and 0.004 to 0.047, respectively; and in Imeruli cheese 0.035, 0.886, 0.013, 0.011, 1.003 and 0.289 mg/kg ww, and Sulguni cheese 0.079, 2.348, 0.03, 0.026, 3.06,and 0.401 mg/kg ww, respectively (Figure 5).

Environmental pollution, especially human-induced pollution, is one of the most significant factors in the occurrence of many trace and toxic elements, along with the phenomena of interelement interactions. For this reason, the data available differs from the permissible limit for these elements to be present in milk and milk products [82].

The selenium concentration in milk varies according to the natural soil content of Se or by geographical location. Selenium is present in soil inorganic salts, where plants absorb these salts, convert them into organic forms of the element (mostly as selenomethionine), and then incorporate them into proteins. In this way, selenium enters the food chain, as dependent mainly on the properties of the soil. There are several reasons for increases of this element in milk, for example, cattle feed supplementation with organic forms of Se (such as selenomethionine [64,83,84,85,86,87], or from the application of inorganic forms of selenium parenterally [88,89]).

The concentration of selenium in all milk samples in this study (Table 1) did not exceed the permissible limit compared with the European Food Safety Authority (0.5 mg/kg) [71]. Compared with other literature sources, the content of Se in the raw milk samples was lower than the values reported by Hermansen et al. (0.0223 mg L^−1^) [90] and Hurle (0.04 mg L^−1^) [67].

As for the Se in the cheese samples, Table 2 shows that both types of cheese, Imeruli and Sulguni, exceeded the limit of Se in the literature 1.003 and 3.06 mg/kg wet weight, respectively. The proportion found in Sulguni cheese was higher than in Imeruli cheese because more milk is needed for this type of cheese.

In addition, the results in Table 1 and Table 2 show manganese levels in milk and cheese. Due to the lack of studies on manganese in milk and cheese, these results are compared with several pieces of literature. According to Knowles et al. [64], the manganese concentration in the milk must be between 0.02 and 0.05 mg L^−1^. Milk samples in 7 villages were within this limit, except for Chapala and Vanati, where they exceeded the permissible limit of 0,075, 0,079 mg L^−1,^ respectively.

In comparison to the permissible limit according to Flynn and Hurley [63,67] (0.03, 0.02 mg L^−1^), the milk samples in this study have exceeded these limits. As for the cheese samples of Imeruli and Sulguni, we find that Mn in all samples exceeded the permissible limit compared to Knowles, Flynn and Hurley [63,64,67]. Additionally, compared to other countries such as Poland, the permitted limit for manganese was 0.102 mg L^−1^ [89], as the samples in this study did not exceed this limit except for Sulguni cheese 2348 mg/kg wet weight.

The amount of manganese in high doses seriously affects human health. It leads to emotional and mental disturbances (lack of coordination and muscle stiffness) and causes impairment of neuromuscular and neurological control [90]. The consumption of milk that contains high doses of manganese that have exceeded permissible limits leads to congenital disabilities, impaired bone development and causes impairment in male fertility. The brain is also very sensitive to an overabundance of manganese, and it is particularly vulnerable to this excess [90].

As the results showed in Table 1, the amount of Chromium and Molybdenum in raw milk was, according to Flynn [63], less than the permissible limit (0.02, 0.05 mg L^−1^), respectively. Likewise, according to Hurley [67], the ratio of these two components in this study did not exceed the permissible limit (0.015, 0.07 mg L^−1^).

As for the Cr and Mo analysis results in cheese samples, Table 2 shows that Chromium and Molybdenum exceeded the permissible limit in Imeruli and Sulguni cheeses (0.035, 0,079 mg/kg), (0.289, 0.401 mg/kg), respectively. However, according to Qin et al. [91], the permissible amount of chromium should not exceed 0.3 mg/kg.

Molybdenum and Chromium are essential trace elements, where molybdenum enters in a cofactor (molybdopterin) for some enzymes, which stimulates oxidation and reduction reactions. There are no sufficient studies or information that show toxicity or problems caused by an excess of Chromium beyond the permissible limit, as most of the experiments are based on animals [92,93]. Chromium is considered non-toxic if taken orally in a normal proportion, as it becomes 100% reduced in the gut. However, high doses of chromium, especially when exposed through the respiratory system, may cause sinonasal cancer [94].

As for nickel, its concentration was lower than the permissible limit in milk and cheese (0.027 mg L^−1^) [63,67]. An excess of Ni over the permissible limit may lead to neurotoxicity, impairment of the male reproductive system [95,96] and oxidative stress [97].

Increased content of cobalt was found in Imeruli and Sulguni cheese samples (0.013, 0.03 mg/kg/ wet weight), While its concentration in milk was less than the permissible limit (0.006 mg L^−1^) [63,66,67]. The increase of cobalt in milk and cheese is directly related to the characteristics of the metabolic processes in a cows’ body, where some metals (such as cobalt) can be transferred directly from the blood into the milk. Cobalt is considered to have a toxic effect if it is consumed at higher than the permissible limit. It may cause central nervous system dysfunction, thyroid disorder and polycythemia [66,98,99].

### 3.3. The Differences in the Presence of Minerals and Trace Elements in Cheese and Milk

A *t*-test was used to compare the trace element content of Imeruli and Sulguni cheese (see Table 3, Table A1). The *t*-test showed a strong significant difference in the content of Imeruli cheese and Sulguni cheese for chromium and selenium. The presence of each of these two elements was higher in Sulguni cheese than in Imeruli cheese, (**Cr**: Mean _Imeruli_ = 0.035, Mean _Sulguni_ = 0.079, *t*= 2.902, *p* < 0.01), (**Se**: Mean _Imeruli_ = 0.878, Mean _Sulguni_ = 3.06, *t* = 2.627, *p* < 0.01).

According to Cohen’s d [100], this effect appears significantly (Cohen’s d _Cr_ = 1.209, Cohen’s d _Se_ = 1.095) and suggests a large effect size of the relevant test.

In addition, these differences appear in both cheese types for each of the following elements: (**Mn**: Mean _Imeruli_ = 0.887, Mean _Sulguni_ = 2.35, *t* = 2.468, *p* < 0.05), (**Pb**: Mean _Imeruli_ = 0.122, Mean _Sulguni_ = 0.259, *t* = 2.229, *p* < 0.05), (**Co**: Mean _Imeruli_ = 0.014, Mean _Sulguni_ = 0.030, *t* = 2.210, *p* < 0.05), (**Cu**: Mean _Imeruli_ = 1.26, Mean _Sulguni_ = 2.46, *t* = 1.936, *p* < 0.05), (**Ni**: Mean _Imeruli_ = 0.005, Mean _Sulguni_ = 0.018, *t* = 1.832, *p* < 0.05). whereas Cohen’s d suggests a large effect size of the relevant test [100]. (Cohen’s d _Mn_ = 1.028, Cohen’s d _Pb_ = 0.092, Cohen’s d _Co_ = 0.921, Cohen’s d _Cu_ = 0.807, Cohen’s d _Ni_ = 0.763).

Thus, these results show that the content of Sulguni cheese from Cr, Se, Mn, Pb, Co, Cu and Ni is higher than Imeruli cheese, which could be due to the use of Imeruli cheese as a basis for making Sulguni cheese or the different production methods [54,55,56,57].

On the other hand, Table 4 and Table A2 shows the difference in the presence of trace elements in milk samples according to their presence in the areas of Mashavera and Khrami rivers. The *t*-test showed a strong significant difference for the presence of manganese and iron in the two rivers. The presence of these two elements in milk samples taken from the Mashavera River region was higher than in the samples taken from the Khrami River region. (**Fe**: Mean _Mashavera_ = 2.493, Mean _Khrami_ = 0.855, *t* = 4.118, *p* < 0.01), (**Mn**: Mean _Mashavera_ = 0.053, Mean _Khrami_ = 0.037, *t* = 2.876, *p* < 0.01).

Cohen’s d value (Cohen’s d **_Fe_** = 0.702, Cohen’s d **_Mn_** = 0.490) suggests a large effect size of the relevant test [100].

The *t*-test (Table 4) also shows a significant difference between copper, lead and chromium in milk samples according to the rivers: (**Cu**: Mean _Mashavera_ = 0.287, Mean _Khrami_ = 0.202, *t* = 2.007, *p* < 0.05), (**Pb**: Mean _Mashavera_ = 0.009, Mean _Khrami_ = 0.006, *t* = 1.749, *p* < 0.05), (**Cr**: Mean _Mashavera_ = 0.003, Mean _Khrami_ = 0.003, *t* = 1.707, *p* < 0.05). Whereas Cohen’s d suggests a large effect size of the relevant test [100]. (Cohen’s d _Cu_ = 0.342, Cohen’s d _Pb_ = 0.298, Cohen’s d _Cr_ = 0.291).

Therefore, these results show more trace elements in milk samples taken from the villages where the Mashavera River is located than the milk samples taken from the villages whose agriculture and irrigation depend on the Khrami River.

One explanation could be that mining companies are more present in the areas of the Mashavera River, as discussed in previous studies [34,36,39,41,42].

## 4. Conclusions

In conclusion, we hypothesized that milk and cheese samples would be contaminated above permissible limits because of soil and water pollution in areas contaminated with heavy metal from the mining industry in Georgia. The results showed that most of milk samples contained heavy metal below the permissible limit compared to the European Union and other literature. However, these results exclude lead from seven samples in each of the following villages: Kvemo Bolnisi, Bolnisi, Mitskineti and Ratawani, where the concentration of lead in some samples was higher than the permissible limits mentioned in the literature and compared to the concentrations stipulated by Georgian laws. The lead concentration in samples taken from these villages was below the permissible limit. As for copper, the amount in the villages of Sabereti and Vanati was above the permissible limits according to the European Union permissible limit.

The results remain the same until the milk is processed to make cheese. Most of these heavy metals exceeded the permissible limits (Cu: 1000 mg/kg ww, Zn: 5000 mg/kg ww, Cd: 0.2 mg/kg ww, Pb: 0.500 mg/kg ww. According to Georgian law, the presence of cadmium and lead was below the permissible limit but compared to the European Union and the other literature, their concentrations were above the permissible limits. As for lead, the results showed that both types of cheese exceeded the permissible limit (according to the European Union). Furthermore, the concentration of Pb in Sulguni cheese was higher than Imeruli cheese due to how this type of cheese is manufactured. As mentioned earlier, Imeruli cheese is used as the basis for making Sulguni cheese. 

Copper concentration in cheese was below the permissible limit according to the Georgia laws. The presence of Co and Ni in cheese samples was also below the permissible limit compared to Cr, Co, Mn and Se, which exceeded these limits. The concentration of all these elements was higher in Sulguni cheese than in Imeruli cheese. Therefore, it is important to note that the presence of heavy metals in Sulguni cheese was high in all samples compared to Imeruli cheese, which could be the subject of future research to determine whether the cheese production causes this.

It is also worth noting that the permissible limits for lead, cadmium, zinc and copper, according to the laws stipulated in Georgia, are considered high when compared with the internationally permissible limits. As Georgia is currently seeking to enter the European Union, therefore following the standards EU regulation is a matter that must be changed, to reach European food safety standards.

This region is considered one of the most important regions in Georgia for smallholder dairy production. However, our results show that milk does not pose any serious health risks to consumers compared to cheese. Despite all this, periodic and regular monitoring of this region is vital and necessary to monitor the presence of heavy metals which might lead to adverse health effects such as immune deficiency.

## Figures and Tables

**Table 3:** Independent Samples *t*-test of the trace elements in Sulguni and Imeruli cheese.

	t	df	*p*	VS-MPR *	Mean Difference	SE (Mean Difference)	Effect Size (Cohen’s d)
Cr	2.902	23	0.004	16.591	0.044	0.015	1.209
Mn	2.468	23	0.011	7.566	1.461	0.592	1.028
Fe	1.024	23	0.158	1.261	32.030	31.290	-
Co	2.210	23	0.019	4.949	0.016	0.007	0.921
Ni	1.832	23	0.040	2.859	0.013	0.007	0.763
Cu	1.936	23	0.033	3.296	1.202	0.621	0.807
Zn	1.642	23	0.057	2.250	48.973	29.828	-
Se	2.627	23	0.008	9.990	2.182	0.830	1.095
Mo	1.574	23	0.065	2.080	0.112	0.071	-
Cd	1.489	23	0.075	1.893	0.002	0.001	-
Pb	2.229	23	0.018	5.103	0.137	0.062	0.929

For all tests, the alternative hypothesis specifies that group Imeruli is less than group Sulguni. Student’s *t*-test. * Vovk-Sellke Maximum *p* -Ratio: Based on a two-sided *p*-value, the maximum possible odds in favour of H₁ over H₀. equals 1/(−e *p* log( *p* )) for *p* ≤ 0.37 [101].

**Table 4:** Independent Samples *t*-Test of the trace elements in milk according to the rivers.

	t	df	*p*	VS-MPR *	Mean Difference	SE Difference	Cohen’s d
Cr	1.707	189	**0.045**	2.646	0.0006	0.0004	0.291
Mn	2.876	189	**0.002**	26.838	0.017	0.006	0.490
Fe	4.118	189	**<0.001**	1232.574	1.638	0.398	0.702
Co	1.383	189	0.084	1.767	0.002	0.001	-
Ni	0.770	189	0.221	1.103	0.0007	0.0009	-
Cu	2.007	189	**0.023**	4.225	0.085	0.042	0.342
Zn	1.226	189	0.111	1.509	0.336	0.274	-
Se	0.083	189	0.467	1.000	0.0002	0.003	-
Mo	-0.016	189	0.506	1.000	−0.0005	0.003	-
Cd	−0.206	189	0.582	1.000	−0.0003	0.0001	-
Pb	1.749	189	**0.041**	2.812	0.003	0.002	0.298

Note. For all tests, the alternative hypothesis specifies that group Khrami is less than group Mashavera. Student’s *t*-test. * Vovk-Sellke Maximum *p* -Ratio: Based on a two-sided *p*-value, the maximum possible odds in favor of H₁ over H₀ equals 1/(−e p log(*p*)) for *p* ≤ 0.37) [101].

**Figure A1:**
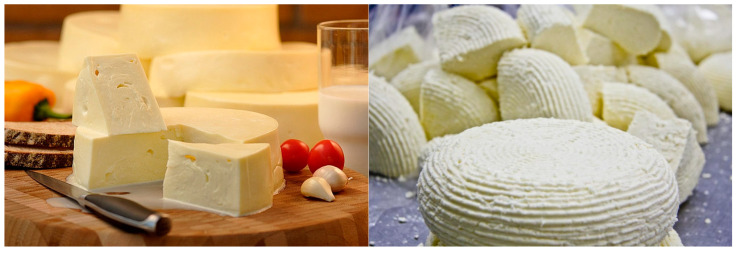
Imeruli and Sulguni cheese, respectively (Source: https://georgianjournal.ge/georgian-cuisine/32138-georgia-among-top-10-countries-on-the-world-cheese-map.html, accessed date: 11 September 2021).

## Data Availability

The datasets generated for this study are available on request to the corresponding author.

## Appendix A

**Table A1:** Descriptive statistic of the trace elements in Sulguni and Imeruli cheese.

Group Descriptives	Group	N	Mean	SD	SE
**Cr**	Imeruli	16,00	0.035	0.017	0.004
	Sulguni	9,00	0.079	0.057	0.019
**Mn**	Imeruli	16,00	0.887	0.595	0.149
	Sulguni	9,00	2,35	2,27	0.756
**Fe**	Imeruli	16,00	69,10	64,92	16,23
	Sulguni	9,00	101,13	91,17	30,39
**Co**	Imeruli	16,00	0.014	0.011	0.003
	Sulguni	9,00	0.030	0.026	0.009
**Ni**	Imeruli	16,00	0.005	0.007	0.002
	Sulguni	9,00	0.018	0.027	0.009
**Cu**	Imeruli	16,00	1,26	0.739	0.185
	Sulguni	9,00	2,46	2,32	0.772
**Zn**	Imeruli	16,00	75,86	52,53	13,13
	Sulguni	9,00	124,84	97,78	32,59
**Se**	Imeruli	16,00	0.878	0.906	0.227
	Sulguni	9,00	3,06	3,14	1048,00
**Mo**	Imeruli	16,00	0.290	0.111	0.028
	Sulguni	9,00	0.401	0.245	0.082
**Cd**	Imeruli	16,00	0.002	0.002	0.0004
	Sulguni	9,00	0.004	0.005	0.002
**Pb**	Imeruli	16,00	0.122	0.093	0.023
	Sulguni	9,00	0.259	0.215	0.072

**Table A2:** Descriptive statistic of the trace elements in milk according to the rivers.

Group Descriptives					
	Group	N	Mean	SD	SE
Cr	Khrami	45	0.003	0.002	0,0003
	Mashavera	146	0.003	0.002	0.0002
Mn	Khrami	45	0.037	0.020	0.003
	Mashavera	146	0.053	0.037	0.003
Fe	Khrami	45	0.855	0.706	0.105
	Mashavera	146	2.493	2.634	0.218
Co	Khrami	45	0.002	0.003	0.0004
	Mashavera	146	0.004	0.008	0.0006
Ni	Khrami	45	0.005	0.004	0.0006
	Mashavera	146	0.006	0.006	0.0004
Cu	Khrami	45	0.202	0.269	0.040
	Mashavera	146	0.287	0.242	0.020
Zn	Khrami	45	3.024	1.194	0.178
	Mashavera	146	3.361	1.714	0.142
Se	Khrami	45	0.014	0.016	0.002
	Mashavera	146	0.015	0.017	0.001
Mo	Khrami	45	0.023	0.019	0.003
	Mashavera	146	0.023	0.019	0.002
Cd	Khrami	45	0.0003	0.001	0.0001
	Mashavera	146	0.0003	0.0007	0.0006
Pb	Khrami	45	0.006	0.007	0.001
	Mashavera	146	0.009	0.010	0.0008
